# Multiple-binding-site mechanism explains concentration-dependent unbinding rates of DNA-binding proteins

**DOI:** 10.1093/nar/gkt1327

**Published:** 2014-01-06

**Authors:** Charles E. Sing, Monica Olvera de la Cruz, John F. Marko

**Affiliations:** ^1^Department of Materials Science, Northwestern University, 2220 Cook Dr. Evanston, IL 60208, USA, ^2^Department of Molecular Biosciences, Northwestern University, Evanston, IL 60208, USA and ^3^Department of Physics and Astronomy, Northwestern University, Evanston, IL 60208, USA

## Abstract

Recent work has demonstrated concentration-dependent unbinding rates of proteins from DNA, using fluorescence visualization of the bacterial nucleoid protein Fis [Graham *et al.* (2011) (Concentration-dependent exchange accelerates turnover of proteins bound to double-stranded DNA. Nucleic Acids Res., 39:2249)]. The physical origin of this concentration-dependence is unexplained. We use a combination of coarse-grained simulation and theory to demonstrate that this behavior can be explained by taking into account the dimeric nature of the protein, which permits partial dissociation and exchange with other proteins in solution. Concentration-dependent unbinding is generated by this simple model, quantitatively explaining experimental data. This effect is likely to play a major role in determining binding lifetimes of proteins *in vivo* where there are very high concentrations of solvated molecules.

## INTRODUCTION

The kinetics of DNA–protein interactions control all aspects of gene expression and therefore cell function, and are of strong biological and biophysical interest. Sequence-targeting proteins such as transcription factors are known to have rapid site-targeting abilities, and the underlying dynamics of target search have been intensely investigated (1–8). These mechanisms generally rely on the existence of nonspecific binding, a property of a wide variety of DNA-binding proteins (1,9,10).

However, much less attention has been paid to unbinding of proteins from DNA. Removal and renewal of proteins bound to DNA is equal in importance to binding in determining transcription states and therefore cell behavior. Recent experimental studies of the proteins Fis (9,11–13), NHP6A (10) and others (14,15) have used novel single-molecule methods to probe unbinding dynamics. These *in vitro* experiments directly visualize the presence of binding proteins using fluorescence microscopy or DNA force spectroscopy measurements (16–21), and have demonstrated an unexpected concentration-dependent off-rate 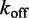, of the form (16):
(1)
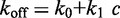

where *k*_0_ is the bare zero-concentration reaction constant and *k*_1_ is a coefficient that incorporates a linear dependence of off-rate to concentration *c* (16). This has been shown to occur for Fis, the main focus of this manuscript, as well as for the DNA-binding proteins HU and NHP6A (16). This suggests that concentration-dependent dissociation may transcend protein-specific secondary structure and be characteristic of more generic tertiary- or quaternary-level features. Similar unbinding behavior has been invoked to understand kinetic data for other DNA-binding proteins (22,23), often in conjunction with other concentration-dependent effects for purposes of characterizing three-body and higher-order interactions (i.e. protein-mediated DNA–DNA interactions) (22,24,25). Analogies can also be drawn to classical reaction-rate descriptions of chemical processes, where interplays exist between kinetics and molecular structure (26).

Interpretation of these experimental results is complicated since similar concentration effects are present even in simple noncovalent systems, necessitating a ‘quantitative’ physical picture of concentration-dependence in binding interactions. Investigations on concentration-dependent processes have explored the role of geometric constraints present due to conformational characteristics, in conjunction with work on rebinding effects to understand the sequence-specific ‘searching’ processes that take into account one-dimensional diffusion, DNA hopping and interplay between specific and nonspecific binding (1–3). Nevertheless, concentration effects are pronounced even for linear chains where such processes are negligible and an alternative ‘reaction-limited’ scenario is necessary (16).

We note a key observation regarding the molecular structure of the systems of interest; proteins such as Fis (9,12) and HU and many other DNA-binding proteins all exhibit a characteristic dimeric quarternary structure. Fis, the protein of main interest in this article, binds two adjacent major grooves of the DNA molecule via two helix-turn-helix domains. We will show that dimeric binding sites, and in general any binding interaction with multiple chemical bonds, can naturally lead to bulk-concentration-dependent off-rates.

Our approach is to model the Fis-DNA system with minimal molecular detail such that the observed experimental behavior is obtained, using a coarse-grained simulation and theoretical model that retains (i) the dimeric nature of the binding protein, (ii) the identical nature of each monomeric structure such that binding of each is likewise identical and (iii) the monomers are each fundamental binding structures that themselves do not have concentration-dependent off-rates. This is sufficient to generate an unbinding pathway with competitive binding, and can be described using straightforward numerical and analytical theory to yield quantitatively accurate predictions for behavior observed in the experimental literature. Our results show that structural characteristics and properties of the environment may be necessary to describe biomolecule dynamics even in straightforward *in vitro* experiments, and suggest that the principles we explore are likely important to understanding a wide range of receptor–ligand and protein–protein interactions.

## MATERIALS AND METHODS

**Figure 1.**
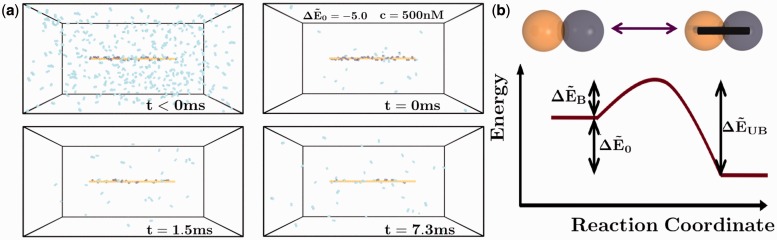
(**a**) Geometry for simulations of DNA chain (orange) in a box with periodic boundary conditions (indicated by black box). At 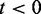 ms, high concentrations of binders (cyan) are introduced and irreversibly bind to the chain. At *t* = 0 ms, bound beads are tagged (dark blue) and cyan binders are removed to attain a concentration *c*. When tagged dimers pass through the boundaries, the monomers become untagged. The total sum of tagged binder monomers is *n_B_*, which decays over time (dark blue binders are less at 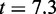 ms than 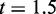 ms). (**b**) Phenomenological energy landscape based on the Bell model, where there is an energy barrier to binding given by 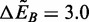 (to yield binding time on the order of the diffusion time 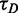) and an energy barrier to unbinding 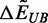. The overall binding energy is given by 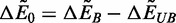.

To investigate the binding kinetics and equilibrium behaviors of dimeric protein binders interacting with a DNA substrate, we use a coarse-grained model that constitutes a minimal representation of the geometric situation and the hypothesized binding energy landscape. These simulations are designed to reproduce the experimental procedures in Graham *et al.* (16), which considers fluorescently tagged Fis molecules, which are initially saturated along a DNA chain that is stretched almost completely using a magnetic tweezers force spectroscopy setup. A linear DNA chain is thus represented in a Brownian Dynamics simulation as 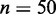 beads that are spatially fixed and can bind exclusively and reversibly with dimeric binders that are free to undergo Brownian motion (simulation snapshots shown in Figure 1a). Binders are represented as dimers of beads, which can each bind independently to the DNA with a Monte Carlo update procedure that reproduces the statistics expected from a traditional Bell Model-type reaction (schematically demonstrated in Figure 1b) (27,28). The binding landscape in Figure 1b is determined by the binding barrier 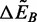 and unbinding barrier 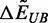, such that there is an energy change on binding of 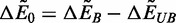 (tildes represent values normalized by 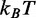). 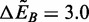 is kept constant, and leads to a binding time 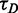 that is equal to the diffusive time of the bead 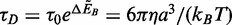, where η is the solvent viscosity and 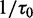 is the Monte Carlo update frequency in the simulation. The characteristic unbinding time for a binding interaction is, thus, 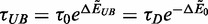. The dimension of the DNA and binder beads have a radius *a*, and the overall box has a size 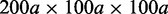. This setup is shown in Figure 1a, which also demonstrates the time evolution of the system. This time evolution proceeds in a fashion analogous to the experimental case; initially, high concentrations of binders that strongly bind to the chain are used to saturate the DNA chain with bound dimers. Bound dimers are ‘tagged’ at *t* = 0 and the external concentration of binders is decreased to the measurement value of *c*. These untagged dimers slowly exchange with the tagged dimers, and once a tagged dimer passes through the periodic boundary conditions it becomes untagged. At long times, the number of tagged dimers decreases as shown in Figure 1a. We provide a detailed methods section in the Supplementary Data (SD) for this manuscript.

## RESULTS AND DISCUSSION

### Equilibrium behavior

While there are nontrivial dynamical behaviors for the system, the equilibrium behavior of these models is analytically solvable by straightforward-statistical mechanical methods. These computations also provide insight into the origins of the binding dynamics for these systems, which we will demonstrate in the context of correlations.

**Figure 2.**
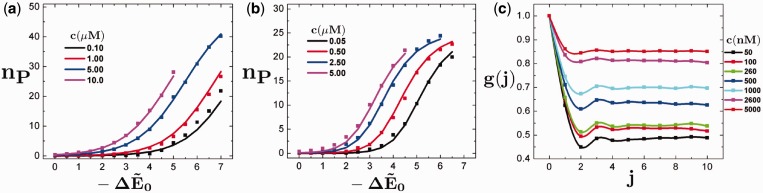
(**a**) Titration curves demonstrating the equilibrium number of binders *n_P_* along a chain of 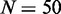 binding sites versus the change in energy on binding 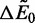 for the monomer (a) and dimer (**b**) situations for a number of different concentrations *c*. Points represent values determined from simulation, and the lines are the theoretical predictions assuming 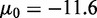 for (a) and 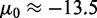 for (b). This demonstrates that the equilibrium behavior of these systems can be described using straightforward statistical mechanics. In (**c**), the along-the-chain binding correlation function *g*(*j*) is plotted versus index distance *j*. At low concentrations (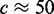 nm) there is an abundance of binders at *j* = 1 due to the dimeric behavior of the binders, along with a correlation hole at *j* = 2. This oscillation becomes smaller at large *c* due to the stabilization of singly bound states. 
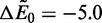
.

We plot the equilibrium properties of the binding equilibrium, as the number of occupied binding sites *n_B_* as a function of the binding energy 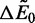 for a number of concentrations *c*. This is plotted in Figure 2a and b, which demonstrates *n_B_* versus 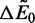 for both monomers (a) and dimers (b). This permits comparison between both simulation and theory, but also illustrates the effect of dimerization constraints on the equilibrium structure. In the monomer case, the well-known adsorption 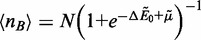 result is plotted as lines in Figure 2a, and fits the data almost exactly. 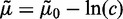 is the ideal gas chemical potential with a constant reference chemical potential 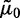. Clearly, the system in Figure 2a reproduces the correct statistical mechanics for the simplified case on varying both *c* and 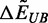. A slightly more complicated transfer matrix calculation described in the SD can be used to determine the analogous result for a dimeric system:
(2)
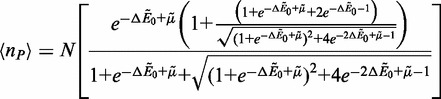

Above we introduce *n_P_*, the number of bound ‘proteins’. This calculation provides the lines demonstrated in Figure 2b, and fits to the data are once again achieved. There is a marked decrease in the location of the apparent transition to a highly bound chain when binders are dimeric, due primarily to binding cooperativity. To further examine the equilibrium structure, we introduce the distinction between singly and doubly bound binders, the former representing states where a binder is only bound at a single monomer with the other monomer unbound and the latter representing the state where both monomers are simultaneously bound.

The singly bound state manifests itself in the along-the-chain binding correlation functions along the DNA substrate. The presence of a binder necessarily limits the binding possibilities of an adjacent binder, which is prevalent in the limit that the binders are primarily doubly bound. We quantify this effect of cooperative binding through the calculation of correlation functions 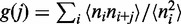, which calculates the probability of having an occupied state 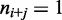 that is *j* binding positions away from an occupied state 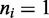. 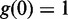 at the *j* = 0 position, so this is equivalent to choosing an already bound monomer at *j* = 0 and looking at the probability of having a bound monomer *j* positions away. This is shown in Figure 2c, which plots *g*(*j*) versus *j* for a number of concentrations *c*. At very low concentrations, there is a large likelihood (compared with the large *j* limit) that there will be binding at the immediately adjacent site. This is due to the dimeric nature of the binders, and simply implies that if one binder bead is bound the adjacent binder bead is likely to be bound at the adjacent site along the DNA.

However, after this binder, there is then a ‘correlation hole’ where the probability of finding a bound binder is significantly smaller. This is due to the presence of the original binder; the large preference for the original dimer to take positions *i* and *i* + 1 limits the possibilities for a dimer at *i* + 2 (it is unlikely to bind at *i* + 1 due to the original dimer). This is a manifestation of the well-known ‘overlap’ effect that provides an anti-cooperative binding behavior for multimeric DNA-binding proteins (29). The resulting oscillations in the correlation function quickly reach their averaged values at 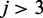. At higher concentrations *c*, these oscillations are suppressed even at low values of *j*. *j* = 1 at large *c* is essentially equivalent to the averaged values for distant 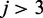 binding sites, suggesting that correlations due to the dimeric structure of the binders are not significant. This suggests the presence of singly bound states, which can ‘fill’ the correlation holes with what we call ‘stabilized singly bound’ states where a singly bound dimer at *i* is immediately adjacent to other binders occupying *i* + 1 and *i* − 1 and thus cannot become doubly bound. Evidence for such singly bound states has been seen experimentally in stoichiometric investigations on Fis binding proteins, where overloading of the DNA can occur beyond the saturation of the chain with doubly bound states (11).

This stabilization of singly bound dimers due to an abundance of neighboring dimers drives nontrivial binding dynamics in these systems. Stabilized singly bound states are much more kinetically accessible to unbinding than doubly bound dimers, allowing for a quicker mechanistic pathway toward unbinding. We analyze this aspect by probing the exchange kinetics of such systems in a way that reflects experimental literature.

### Exchange kinetics

Experiments on DNA/protein binding using fluorescently tagged proteins to facilitate direct visualization of protein exchange are reproduced in simulation, as described by the ‘Simulation Methods’ section, which outlines a protocol meant to directly correspond to experimental procedure (16,17). We begin with 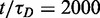 of irreversible binding with a high concentration of binders to the DNA beads. This drives the system into a state where the chain is saturated with binders (mostly doubly bound). The concentration of surrounding (unbound) binders *c* is immediately dropped to a set number (anywhere from 4 to 400 dimers in the simulation box). The dimers bound to the chain are tagged. The bound dimers remain tagged until they cross the simulation boundary, whereupon they behave as one of the untagged binders. The beads are not removed, for the sake of simulation simplicity; however, in principle this could be done. This means that, in the upcoming theoretical calculations used for comparisons to simulations, *c* changes as the number of unbound dimers increases during the course of unbinding the initially tagged molecules. Comparisons made to experiment are not affected by this, as the concentration *c* is controlled by the applied flow that removes excess binders.

The number of tagged beads *n_B_* in the simulation starting from 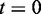 is plotted in a semilog plot in Figure 4a–c as a function of time *t* for a number of different concentrations *c*. The results are averaged over 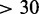 individual trajectories. There is a decay from 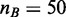 as time progresses, corresponding to the unbinding and subsequent diffusion of binders to the boundaries of the system. Three values of 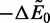 are considered, and drastic differences are obtained. Importantly, as the value of 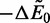 is increased (more binding affinity for the DNA chain), there is a corresponding increase in the effect of external concentration on the system. For small unbinding barriers and low binding affinities 
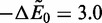
, there is little difference among the various concentrations. This suggests that the rate of unbinding is largely due to the diffusion of binders away from the DNA molecule, which is the rate-limiting step in the process of unbinding and diffusing out of the system. However, for values of 
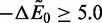
 (Figure 4b and c) the time scale of unbinding drastically decreases on increase of *c*. We have plotted the decay of these molecules on a semi-log plot (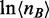 versus *t*) that reveals exponential decay at long time scales. This concentration-dependent exchange is concomitant with an overall decrease in the number of binders 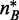 (the asterisk indicates that we consider both tagged and untagged) as the binder-saturated DNA relaxes to equilibrium. We plot this in the Figure 4b inset as the number of binders 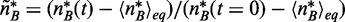 normalized such that it relaxes from 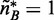 to 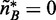, and observe a similar concentration-dependence where higher *c* results in more rapid equilibration.

We note the presence of a small decay that occurs in the first 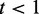 ms of Figure 4b and the first 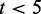 ms of Figure 4c beyond the exponential decay at long times. This is due to the initial departure of a minority species of singly bound dimers that were present in the initial setup before the exchange process, and does not significantly affect our results. Rapid equilibration toward an initial state of near-saturation of doubly bound binders is also expected in experiment, since the experimental setup begins in a concentration regime where singly bound states are abundant and is brought into a regime where doubly bound states are known to prevail (11). The relative speed at which this process would occur is so rapid that it is not expected to be captured within the time resolution of the experimental data, and many of the singly bound binders convert directly into doubly bound binders to fill vacancies created from singly bound departures.

We seek a quantitative description of this concentration-dependent exchange process, and aspire to consider its consequences at values that are relevant to experimental values. Even with these relatively straightforward and coarse-grained calculations, simulations at the time scale of 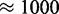 s are prohibitively computationally expensive. Therefore, we develop theory that accurately describes simulation at short time scales and can be extrapolated to experimental time scales of 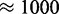 s. To theoretically describe this behavior, we adapt a dynamic scheme to model the evolution of the system during an exchange experiment.

### Exchange kinetics theory

**Figure 3.**
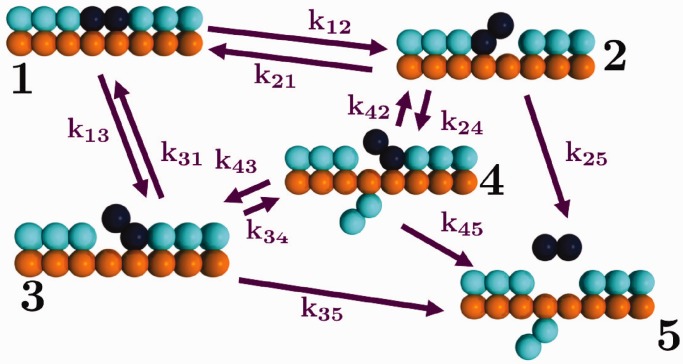
Schematic demonstrating the states used in the theoretical model for the dynamics of dimer-DNA binding. State 1 is a fully bound dimer (dimer is blue, DNA chain is orange, other binders are cyan), which is the initial state of the binders at the onset of an exchange experiment. States 2 and 3 are singly bound dimers with an open and adjacent DNA binding site. In State 4, this adjacent DNA binding site is occupied by a second dimer that has bound from the previously unbound binder population. The dangling side of the binder is therefore not able to rebind and revert to State 1, which is possible in 2 and 3. States 2, 3 and 4 can subsequently unbind their last remaining side to unbind completely from the DNA chain (State 5). Pathways between the States *i* and *j* are indicated with appropriate rate constants *k_ij_*.

The articulation of these states suggests the use of a Master Equation approach, which has been demonstrated to be successful in describing similar situations where discrete changes in state occur on binding and unbinding (30). The Master Equation is a generalized version of a classical rate reaction equation, and is related to other dynamical equations such as the Fokker-Planck and Langevin equations (31). We write the Master Equation as 
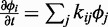
 where 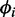 is the set of states indexed by *i* and *k_ij_* is the generalized rate constant for the motion of *j* to *i*. We define the matrix *k_ij_* using the state map shown in Figure 3, which articulates a number of states that represent our definition of 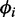. 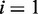 is the situation that the binder is fully bound to the DNA, 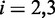 are states where the binder has separated on one of the monomers, leaving an open spot that is unbound, 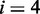 occurs when another binder replaces that open spot, and 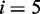 occurs when both binders have unbound. The connections between these states form the map in Figure 3, where each arrow corresponds to a value in the definition of a matrix *k_ij_*. The end result is a 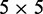 matrix that is based on the conceptually relevant motions between states that is based on the probabilities used in the Bell model aspect of the simulation. Three types of transitions occur: for a fixed time interval 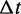 set by the Monte Carlo aspect of the simulation, unbinding transitions occur with a probability 
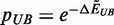
, binding transitions from a half-bound dimer occur with a probability of 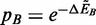 and binding transitions with a unbound dimer occur with a probability 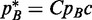, where *C* is a constant that accounts for the geometric proportionality between the number concentration *c* and the likelihood that a free dimer will have a binding opportunity over 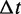. For our calculations, we use 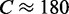; this is related to the volume within which a dimer can be located and be able to bind to the DNA binding site, and is given by 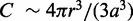. The ideal value is 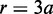 (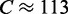), which is the distance between the binding site center and the dimer center if all three are lined up and adjacent. We find a slightly larger value (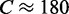 corresponding to 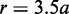) provides a better fit to the data, which may be due to excluded volume effects of the chain or details of the association mechanism used in our simulations.

**Figure 4.**
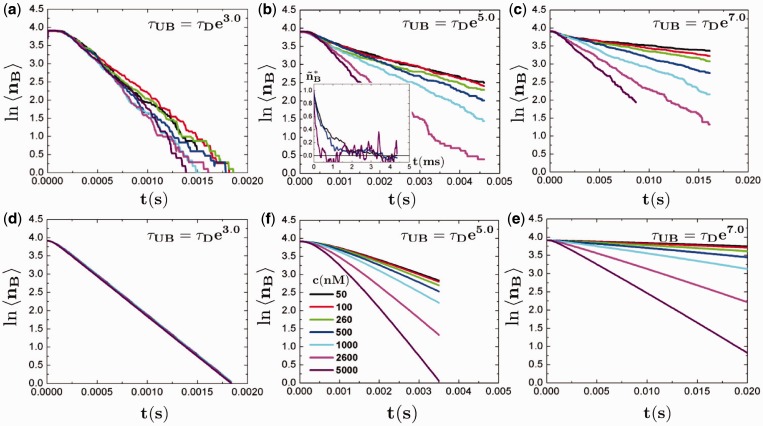
Binder exchange simulation data for (**a**) 
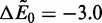
, (**b**) 
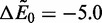
 and (**c**) 
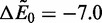
. At time 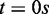, the DNA chain is saturated with binders (number of occupied sites 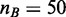), and the concentration [shown in legend in (b)] is immediately applied to the system. In (a) the exchange is mostly diffusion-limited, and there is not a large difference on changing the binder concentration. In (b) and (c) exchange occurs at different rates depending on the external concentration *c* of untagged binders. Numerical calculation using the matrix *k_ij_* in Equation (3) yields nearly quantitative matching, as shown in (**d–f**) [corresponding to (a–c), respectively]. The inset in (b) plots the normalized overall relaxation of the number of tagged and untagged binders 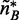 as a function of time *t* in analogy to the main plot in (b). Colors correspond to concentrations in b and demonstrate a concentration-dependent approach to equilibrium (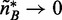) that is concomitant with the concentration-dependent exchange process.

Based on the transition probabilities *p_UB_*, *p_B_* and 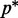, along with the state map in Figure 3, we write the system evolution matrix *k_ij_* as follows:
(3)
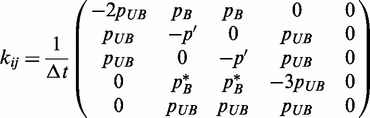

where nonexistent arrows in Figure 3 are 0, 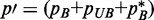, and 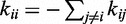 ensures that the distribution 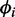 among states *i* remains properly normalized. It is possible to numerically use this matrix to calculate the exchange process directly, a process that we elaborate on in the SD. The results of this numerical calculation are shown in Figure 4d–f alongside the simulation data, and demonstrate that there is near-quantitative matching between the two approaches for all the values of *c* and 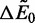. This significantly illustrates the effectiveness of this coarse-grained approach in describing what is suspected to be a general phenomenon; a dimeric protein with monomeric portions that themselves have long-lasting binding behaviors will exhibit a concentration-dependent change due to the stabilization of the singly bound state that is put into our theoretical model via the kinetic description of State 4 in Figure 3.

### Analytical expression for concentration-dependent rate constants

**Figure 5.**
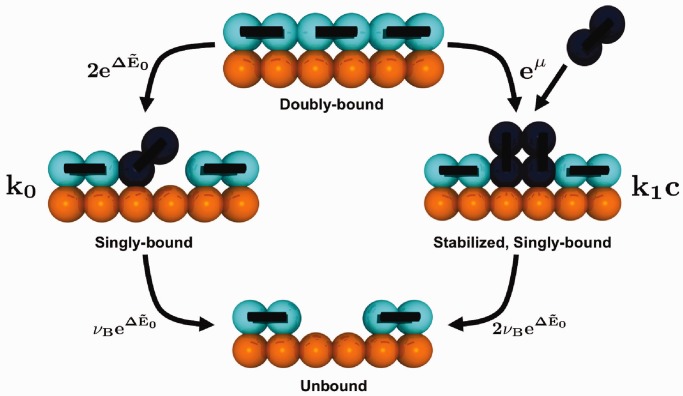
Schematic demonstrating the kinetic pathways used by the theory to develop Equation (4). A doubly bound dimer proceeds either through a concentration-independent pathway (left) to yield 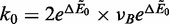, or through the realization of a stable singly bound state (right) to yield 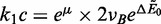.

It is possible to develop an analytical result on incorporation of the above scheme into a conceptual understanding of the numerical results. We define an effective off-rate constant 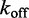, and use an assumption that the ‘excited’ singly bound states are in equilibrium with the doubly bound state and that overall unbinding results from the unbinding of a singly bound state only. This is therefore succinctly defined as 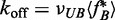 where the value 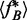 corresponds to the fraction of bound dimers that are singly bound (and can therefore separate from the main chain). There are two different subpopulations within this singly bound fraction, schematically represented in Figure 5: binders that are ‘excited’ from the doubly bound state and can readily rebind owing to the presence of an adjacent unbound DNA site (denoted with a 0 subscript), and binders that are stabilized in a singly bound state due to the immediately adjacent bound dimers preventing rebinding to an unbound DNA site (denoted with a 1 subscript). The former state occurs in the same fashion regardless of the surrounding concentration, due to its origins as a doubly bound chain that is ‘excited’ into the singly bound state with a fraction 
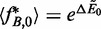
. On unbinding from the singly bound state, the overall pathway represents the bare unbinding constant of the overall binder 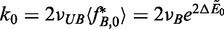 where the unbinding frequency is simply 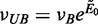 and the factor of two corresponds to the two different paths to unbinding (left monomer first or right monomer first). The stabilized singly bound state is present in larger quantities, since in equilibrium a pair of singly bound dimers can be included without changing the overall binding energy with the DNA (as occurs in the excited singly bound state). This follows from the creation of a pair as in Figure 5 (essentially equivalent to State 4 in Figure 3), which are now both singly bound and long-lasting. A chemical potential equilibrium must occur between the solution and the singly bound pairs (which have a mixing entropy), which we write as 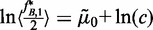 We note the factor of two corresponds to the creation of two singly bound states for each increase in a single molecule bound along the chain, and that there is not a corresponding factor of two in front of the natural log on the left side of the equals sign, as we consider this pair to be located immediately adjacent on the time scales of interest. Using the fraction of stabilized, singly bound dimers 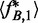 to calculate its contribution to the effective unbinding rate constant 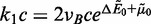 The overall expression for the rate constant is thus given as follows:
(4)
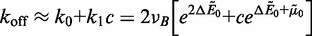

assuming that the two mechanisms described are the most important ones. This is in the form proposed by Graham *et al.* (16) and it leads to a clear physical interpretation that we anticipate will lead to the facile implementation of these results to much more complicated systems. While this picture is (like the more elaborate numerical calculation) kinetic in nature, the key assumption rendering this analytical result is that the stabilized singly bound state is present in a large enough population to be considered in quasi-equilibrium with the ‘bath’ of doubly bound binders.

### Comparison between experimental, analytical and numerical results

**Figure 6.**
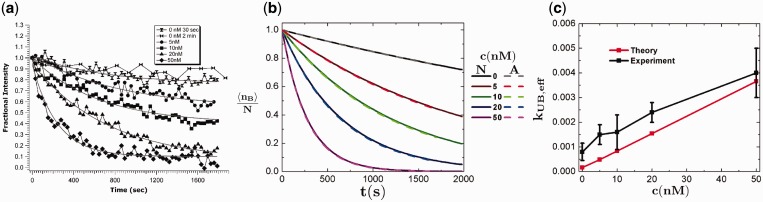
(**a**) Exchange results from the experimental literature [Graham *et al.* (16)] that demonstrate the concentration-dependent decrease in fluorescence due to a change in the unbinding reaction constant (Used by permission of Oxford University Press). (**b**) The analogous exchange calculation (*n_B_* versus time *t*) for a binding energy of 
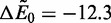
, at concentrations relevant to experiments carried out by Graham, *et al.* (16). Both numerical (N) and analytical calculations (A) are performed, with quantitative matching between the two for 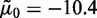 in the analytical result. Qualitative matching of both of these to experimental observations in (a) is observed to occur, suggesting that this model captures much of the physics of concentration-dependent binding dynamics. Effective unbinding rate constants 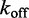 (in 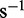) can be extracted from these plots using exponential fits, and the results are in (**c**). Experimental results come from Graham, *et al.* (16). Qualitative matching is demonstrated, and better fits may be obtained on adjustment of both 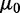 and 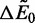 used in the theory.

We can make a direct comparison between results for existing experimental literature and the aforementioned hypothesis for the binding behavior observed in the analytical and numerical results. We note that in Figure 4, near-quantitative matching occurred between numerical and simulation investigations; this established that the appropriate physics are articulated in the numerical calculation such that simulation is reproduced; however, for the time scales of experimental data (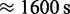) only the numerical calculation is computationally expedient. Figure 6 plots the experimental (a), numerical and analytical (both b) results on plots of bound fraction 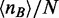 versus time *t* in seconds. A number of concentrations *c* in nM are used, and clearly the appropriate trends are observed. Figure 6 demonstrates the predictions for the experimental and theoretical values for 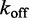 as a function of concentration *c*. The slope is *k*_1_ and the intercept is *k*_0_, both values which match qualitatively with the theoretical predictions. Disparities arise in the apparent equilibrium values of the decays observed in the experiment versus the calculation, with the latter decaying to 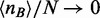 and the former demonstrating a decay to a finite 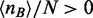. We attribute this to sequence-dependent effects, and verify that this result is likely due to binding energy inhomogeneity, via analysis in the SD of the case of Gaussian random distributed binding energies (Supplementary Data, Fig. S3). The manifestation of this effect suggests that sequence-dependence may richly affect concentration-dependent off-rate behavior in *in vivo* environments where local binder competition may couple with highly variable and correlated sequence-dependent binding affinities.

## CONCLUSION

Experimental work on DNA-binding proteins Fis, HU and NHP6A has recently demonstrated the appearance of concentration-dependent unbinding (16), with similar behaviors observed in other systems (22). This behavior does not occur in usual models of protein–DNA interactions widely used to fit kinetic data where there is by construction a concentration-independent off-rate. This concentration-independent unbinding models a ‘spontaneous dissociation’ pathway, involving a transition over a rate-limiting barrier associated with the DNA–protein complex itself and not dependent on other nearby molecules. Here we have shown that simply generalizing such models to dimeric proteins having *two* interaction sites per protein, with each subunit having concentration-independent unbinding kinetics, leads to ‘concentration-dependent’ off-kinetics of the form 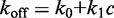 as observed for Fis. This arises from the conformational flexibility of the dimeric protein model, which allows partial dissociation of one side of the protein from its binding site, and subsequent capture of that site by a second protein from solution. The rate at which this can occur is simply controlled by solution phase concentration, as observed in the single-molecule studies (16,22). Our model is able to reproduce rates seen experimentally well, with reasonable choices of microscopic parameters. We note that in the concentration range near to the apparent dissociation point, exchange is the dominant process controlling dissociation, and there is effectively a stabilization of the partially bound state.

Our model is exceedingly simple; undoubtedly more complex behaviors can be generated by addition of additional features. For example, we do not consider the role of binding cooperativity in this model except through the implicit inclusion of overlapping anticooperative effects present in multimeric-binding systems (29). Cooperativity may serve to promote competition effects by favoring the clustering of binders that promotes singly bound dimers. We also consider DNA chains that are essentially saturated; *in vivo* behavior may provide different mechanisms for binding site competition or sequence-dependent effects that are not articulated in this model. These are thus open questions that when investigated may reveal a rich array of competitions that could enhance or suppress concentration-dependent unbinding effects. However, the present article shows that much of the concentration-dependent unbinding observed for Fis can be attributed to its dimeric structure, without invoking more complex causes.

The simplicity of our model suggests that concentration-dependent unbinding effects should be observable for a wide range of molecular interactions. The only essential ingredients are at least two isolated binding elements, and sufficient conformational flexibility of the binding partners so that partial unbinding can allow solvated species the ability to compete with partially unbound ones. We anticipate that the mechanism discussed here for concentration-dependent unbinding may well be applicable to interactions dependent on ‘single’ binding domains, as long as they involve multiple chemical interactions, as is usual even for protein–DNA and protein–protein interactions involving ‘single’ binding-domains [which might be considered to be the case for NHP6A (16)]. Furthermore, the mechanism discussed in this article for acceleration of off-rates may be crucial to turning over proteins on DNA, thus increasing the speed of response for changing gene regulation. One could imagine that many DNA-binding proteins have been selected for their ability to be enhanced by this mechanism: a given protein may be stably bound when suitable competitors are not present, but then may be rapidly replaced by another molecule itself selected to be able to invade the former’s binding site. We anticipate that *in vivo*, where large concentrations of molecules compete for binding partners, the mechanism for concentration-dependent exchange outlined here may strongly effect the rates of turnover of many molecular species through competitions with effects such as cooperativity, sequence dependence and local crowding. However, we also emphasize that as yet, concentration-dependent unbinding has only been quantitatively studied for a few proteins interacting with DNA; careful case-by-case studies are necessary to determine the generality of this phenomenon.

## SUPPLEMENTARY DATA

Supplementary Data are available at NAR Online.

## FUNDING

National Science Foundation [DMR-0907781, MCB-1022117 and DMR-1206868]; the National Institutes of Health [1U54CA143869-01 (NU-PS-OC) and 1R01GM105847-01]; the Office of the Director of Defense Research and Engineering and Air Force Office of Scientific Research [FA9550-10-1-0167]; and the Northwestern International Institute for Nanotechnology. Funding for open access charge: NIH [1U54CA143869-01].

*Conflict of interest statement*. None declared.

## Supplementary Material

Supplementary Data

## References

[1] Halford SE, Marko JF (2004). How do site-specific DNA-binding proteins find their targets?. Nucleic Acids Res..

[2] Bert OG, Winter RB, von Hippel PH (1981). Diffusion-driven mechanisms of protein translocation on nucleic acids. 1. Models and theory. Biochemistry.

[3] Hu T, Shklovskii BI (2006). How does a protein search for the specific site on DNA: the role of disorder. Phys. Rev. E.

[4] Gorman J, Greene EC (2008). Visualizing one-dimensional diffusion of proteins along DNA. Nat. Struct. Mol. Biol..

[5] Lomholt MA, van den Broek B, Kalisch SMJ, Wuite GJL, Metzler R (2009). Facilitated diffusion with DNA coiling. Proc. Natl Acad. Sci. USA.

[6] Elf J, Li G-W, Xie XS (2007). Probing transcription factor dynamics at the single-molecule level in a living cell. Science.

[7] Wang YM, Austin RH, Cox EC (2006). Single molecule measurements of repressor protein 1D diffusion on DNA. Phys. Rev. Lett..

[8] Blainey PC, van Oijen AM, Banerjee A, Verdine GL, Xie XS (2006). A base-excision DNA-repair protein finds intrahelical lesion bases by fast sliding in contact with DNA. Proc. Natl Acad. Sci. USA.

[9] Dorman C (2009). Nucleoid-associated proteins and bacterial physiology. Adv. Appl. Microbiol..

[10] Stillman DJ (2010). Nhp6: A small but powerful effector of chromatin structure in *Saccharomyces cerevisiae*. Biochim. Biophys. Acta.

[11] Skoko D, Yoo D, Bai H, Schnurr B, Yan J, McLeod SM, Marko JF, Johnson RC (2006). Mechanism of chromosome compaction and looping by the *E. Coli* nucleoid protein Fis. J. Mol. Biol..

[12] Shao Y, Feldman-Cohen FS, Osuna R (2008). Functional characterization of the *Escherichia coli* Fis-DNA binding sequence. J. Mol. Biol..

[13] Stella S, Cascio D, Johnson RC (2010). The shape of the DNA minor groove directs binding by the DNA-bending protein Fis. Genes Dev..

[14] McCauley M, Hardwidge PR, Maher LJ, Williams MC (2005). Dual binding modes for an HMG domain from human HMGB2 on DNA. Biophys. J..

[15] Sugimura S, Crothers DM (2006). Stepwise binding and bending of DNA by *Escherichia coli* integration host factor. Proc. Natl Acad. Sci. USA.

[16] Graham JS, Johnson RC, Marko JF (2011). Concentration-dependent exchange accelerates turnover of proteins bound to double-stranded DNA. Nucleic Acids Res..

[17] Graham JS, Johnson RC, Marko JF (2011). Counting proteins bound to a single DNA molecule. Biochem. Biophys. Res. Commun..

[18] Skoko D, Wong B, Johnson RC, Marko JF (2004). Micromechanical analysis of the binding of DNA-bending proteins HMGB1, NHP6A, and HU reveals their ability to form highly stable DNA-protein complexes. Biochemistry.

[19] Skoko D, Yan J, Johnson RC, Marko JF (2005). Low-force DNA condensation and discontinuous high-force decondensation reveal a loop-stabilizing function of the protein Fis. Phys. Rev. Lett..

[20] Xiao B, Johnson RC, Marko JF (2010). Modulation of HU-DNA interactions by salt concentration and applied force. Nucleic Acids Res..

[21] Micah MJ, Rueter EM, Rouzina I, Maher LJ, Williams MC (2013). Single-molecule kinetics reveal microscopic mechanism by which high-mobility group B proteins alter DNA flexibility. Nucleic Acids Res..

[22] Joshi CP, Panda D, Martell DJ, Andoy NM, Chen T-Y, Gaballa A, Helmann JD, Chen P (2012). Direct substitution and assisted dissociation pathways for turning off transcription by a MerR-family metalloregulator. Proc. Natl Acad. Sci. USA.

[23] Ha TJ (2013). Single-molecule approaches embrace molecular cohorts. Cell.

[24] Sidorova NY, Scott T, Rau DC (2013). DNA concentration-dependent dissociation of *Eco*RI: Direct transfer or reaction during hopping. Biophys. J..

[25] Kunzelmann S, Morris C, Chavda AP, Eccleston JF, Webb MR (2010). Mechanism of interaction between single-stranded DNA binding protein and DNA. Biochemistry.

[26] Flory PJ (1953). Principles of Polymer Chemistry.

[27] Bell GI (1978). Models for specific adhesion of cells to cells. Science.

[28] Sing CE, Alexander-Katz A (2011). Equilibrium structure and dynamics of self-associating single polymers. Macromolecules.

[29] McGhee JD, von Hippel PH (1974). Theoretical aspects of DNA-protein interactions: co-operative and non-co-operative binding of large ligands to a one-dimensional homogeneous lattice. J. Mol. Biol..

[30] Sing CE, Alexander-Katz A (2012). Force spectroscopy of self-associating homopolymers. Macromolecules.

[31] Zwanzig R (2001). Nonequilibrium Statistical Mechanics.

