# Supplementary material for: Multiple-binding-site mechanism explains concentration-dependent unbinding rates of DNA-binding proteins
Source: Nucleic Acids Res. 2014 Jan 6;42(6):3783–91. doi: 10.1093/nar/gkt1327 (PMC3973338; doi:10.1093/nar/gkt1327)
Supplement: Supplementary Data [file supp_42_6_3783__index.html]

Multiple-binding-site mechanism explains concentration-dependent unbinding rates of DNA-binding proteins — Supplementary Data 

# Multiple-binding-site mechanism explains concentration-dependent unbinding rates of DNA-binding proteins

## Supplementary Data

files

**Files in this Data Supplement:**

- Supplementary Data - pdf file
