# Supplementary material for: Multiple-binding-site mechanism explains concentration-dependent unbinding rates of DNA-binding proteins
Source: Nucleic Acids Res. 2014 Jan 6;42(6):3783–91. doi: 10.1093/nar/gkt1327 (PMC3973338; doi:10.1093/nar/gkt1327)
Supplement: Supplementary Data [file supp_gkt1327_NAR_CES_02SDJM-final.pdf]

Published online XX XXXX 2013 *Nucleic Acids Research, 2013, Vol. XX, No. XX* **1–6**
doi:10.1093/nar/gkn000

Multiple-binding-site mechanism explains concentration-dependent unbinding rates of DNA-binding proteins

Charles E. Sing [1], Monica Olvera de la Cruz [1,*] and John F. Marko [2,**]

[1] Department of Materials Science, Northwestern University, 2220 Cook Dr. Evanston IL 60208, USA, [2] Department of Molecular Biosciences, Northwestern University, Northwestern University, Evanston IL 60208, USA, and [3] Department of Physics and Astronomy, Northwestern University, Evanston IL 60208, USA

Received January 1, 2013; Revised February 1, 2013; Accepted March 1, 2013

ABSTRACT

Recent work has demonstrated concentration-dependent unbinding rates of proteins from DNA, using fluorescence visualization of the bacterial nucleoid protein Fis (J.S. Graham, et al. Nucleic Acids Research 2011, 39:2249.). The physical origin of this concentration-dependence is unexplained. We use a combination of coarse-grained simulation and theory to demonstrate that this behavior can be explained by taking into account the dimeric nature of the protein which permits partial dissociation and exchange with other proteins in solution. Concentration-dependent unbinding is generated by this simple model, quantitatively explaining experimental data. This effect is likely to play a major role in determining binding lifetimes of proteins *in vivo* where there are very high concentrations of solvated molecules.

SIMULATION METHODS

We use a Brownian Dynamics simulation that incorporates a Monte-Carlo type update step to simulate binding and unbinding events. The former iteratively solves the discretized Langevin equation (with finite time increments δt) to update a number of particles i with a radius of a and positions $\mathbf{r_i}$:

$$\frac{\partial \mathbf{r}_i}{\partial t} = -\sum_j^N \mu_{\mathbf{ij}} \cdot \nabla_j U_{ij} + \xi_i \qquad (1)$$

where $\mu_{\mathbf{ij}} = \delta_{ij}/(6\pi\eta a)$ is the Stokes mobility without the effect of hydrodynamic interactions (though in principle they could be included), $\xi_\mathbf{i}$ is a random velocity satisfying the fluctuation-dissipation theorem ($\langle \xi_\mathbf{i} \rangle = 0$ and $\langle \xi_\mathbf{i} \xi_\mathbf{j} \rangle = 2k_BT\mu_{\mathbf{ij}}\delta_{ij}$), δ_{ij} is the Kronecker delta, and U_{ij} is the pairwise interaction between particles i and j:

$$U_{ij} = k_BT\tilde{\epsilon} \sum_{\alpha\beta ij} \omega^{LJ}_{\alpha\beta ij} \left[\left(\frac{2a}{r_{ij}}\right)^{12} - 2\left(\frac{2a}{r_{ij}}\right)^{6} \right] + \\ + \frac{k_BT}{2a^2}\tilde{\kappa} \sum_{\alpha\beta ij} \omega^{C}_{\alpha\beta ij} \left(r_{ij} - 2a\right)^2 \qquad (2)$$

where the first contribution is a Lennard-Jones potential of strength $\tilde{\epsilon}k_BT$ that occurs between beads i and j a distance r_{ij} apart, and the second contribution is a spring potential with spring constant $\tilde{\kappa} = 200.0$ that provides connectivity between beads i and j. This value of $\tilde{\kappa}$ assures connectivity between i and j such that deviations from a distance of $r_{ij} = 2a$ are small. The matrices $\omega^{LJ}_{\alpha\beta ij}$ and $\omega^{C}_{\alpha\beta ij}$ provide the information regarding interaction and connectivity respectively between beads i and j of type α and β. In our system, we have two groups of beads α/β: DNA beads D and binder beads B. We focus largely on dimeric sets of binders due to the dimeric geometry of most non-specific DNA binding proteins.(1, 2, 3) We use the constraint $\omega^{C}_{BBij} = \omega^{C}_{BBji} = 1$ ($\omega^{C}_{BBij} = 0$ otherwise) when i is even and $j = i+1$ for the dimeric binders. In monomeric situations, $\omega^{C}_{BBij} = 0$ for all binder pairs. We also use the conditions that $\omega^{LJ}_{BDij} = 1$ (binders interact with DNA through Lennard-Jones forces), $\omega^{LJ}_{BBij} = 0$ (binders do not interact through Lennard-Jones), and ω^{C}_{BDij} is a time-dependent manifestation of the connectivity that arises due to binding interactions. This behavior is based on the Bell model of biological interactions,(4) and the following manifestation in Brownian Dynamics is adapted from previous work on biological systems.(5, 6)

The matrix ω^{C}_{BDij}, which we will denote as $\omega_{R,ij}$ for brevity, represents the accounting of all the binding interactions between proteins and DNA monomers. In a nonbonding scenario, $\omega_{R,ij} = 0$ and the harmonic potential in Equation S2 is not applied between the two species. The

*To whom correspondence should be addressed. Tel: +44 000 0000000; Fax: +44 000 0000000; Email: john-marko@northwestern.edu

© 2013 The Author(s)
This is an Open Access article distributed under the terms of the Creative Commons Attribution Non-Commercial License (http://creativecommons.org/licenses/by-nc/2.0/uk/) which permits unrestricted non-commercial use, distribution, and reproduction in any medium, provided the original work is properly cited.

possibility of binding, and consequently unbinding, occurs through a Monte-Carlo type update step that is applied every time interval τ_0. We note that this time interval is chosen such that computational expediency and accurate statistics were obtained, however otherwise the selection is arbitrary; the jump frequency is therefore defined as $\nu_0 = 1/\tau_0$ in the simulation, however in principle the combination of this jump frequency with the energy barrier allows for a redefinition of the absolute energy scale used to describe results. Each time interval τ_0, the matrix is recalculated based on the update step:

$$\omega_{R,ij}(t) = \begin{cases} \begin{cases} 1\ if\ \Xi < e^{-\Delta\tilde{E}_B} \\ 0\ if\ \Xi > e^{-\Delta\tilde{E}_B} \end{cases} & if\ \omega_{R,ij}(t-\tau_0) = 0\ and\ r_{ij} < r_{RXN} \\ \begin{cases} 1\ if\ \Xi < e^{-\Delta\tilde{E}_{UB}} \\ 0\ if\ \Xi > e^{-\Delta\tilde{E}_{UB}} \end{cases} & if\ \omega_{R,ij}(t-\tau_0) = 1 \end{cases} \quad (3)$$

where Ξ is a random number between 0 and 1, chosen randomly for each i and j in every update step τ_0. Energies denoted with tildes are normalized by k_BT (i.e. $\tilde{E}_{UB} = E_{UB}/(k_BT)$; positions and times can also be normalized by the bead radius a and single bead diffusion time $6\pi\eta a^3/(k_BT) = \tau_D$, which when referenced in this manner will also be denoted with a tilde. $r_{RXN}(=2.1a)$ is a reaction radius which sets the spatial limit within which binding can occur. For this simulation, the time step of the Langevin simulation is $\delta t = 0.002\tau_D$ and the Monte Carlo update step is $\tau_0 = 0.05\tau_D$.

What the $\omega_{R,ij}$ matrix conceptually does is it places a spring connection between spatially adjacent beads if they meet the Boltzmann factor-based criterion for overcoming the energy barrier to transition into a bound state (and vice versa). This reproduces the appropriate thermodynamics for a binding pair,(5) and provides a means to change the binding kinetics of a single interaction through changing the unbinding barrier $\Delta\tilde{E}_{UB}$ and the binding energy $\Delta\tilde{E}_0$ independently. In our system, we fix the value of $\Delta\tilde{E}_B$ to reflect the idea that the relevant time scale for binding τ_B is on roughly the same order of magnitude as the binder diffusion. With a value of $\Delta\tilde{E}_B = 3.0$, we reproduce this behavior since $\tau_B = \tilde{\tau}_0 e^{\Delta\tilde{E}_B} \approx 1.0\tau_D$. The binding barrier and binding energy are therefore changed simultaneously, and we report these values in terms of the binding energy $\Delta\tilde{E}_0$.

The overall simulation takes place in a box with periodic boundary conditions $100a \times 100a \times 200a$ with the DNA molecule represented by N beads centered along the long axis of the simulation box (see simulation snapshot in Figure 1). To expedite the simulation and compare it to systems where the DNA is tethered and stretched significantly such that its thermal motions are highly constrained, we do not update the position of the DNA beads in the iteration through the Langevin equation and consider it immobile in our model.

CHAIN-LENGTH DEPENDENCE

One common consideration in the study of DNA/protein interactions is the length and topology of the DNA chain itself,(7, 8) especially since there are hypothesized states where the binding protein is unbound but still affiliated with a nearby DNA chain.(8) Such effects may be due to long-distance chain-chain correlations, and recent work has elucidated the geometric possibility of a random walk escaping from the vicinity of a chain of various dimensions (straight chain, random coil, and collapsed globule) without rebinding.(7) These investigations suggest that rebinding is not strongly dependent on length of a straight DNA segment like the ones in our simulation,(7) however we nevertheless test to see the importance of such effects by adjusting the length of the DNA chain.

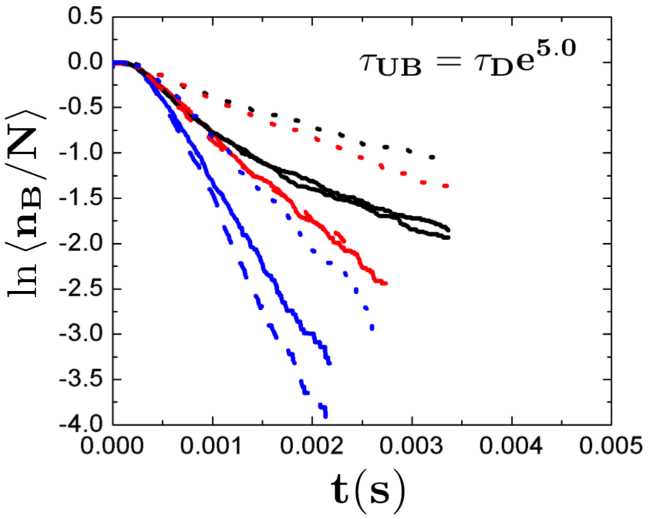

Figure S1. Chain length-normalized exchange kinetics ($\ln\langle n_B/N\rangle$ versus t) for $\Delta\tilde{E}_0 = -5.0$, and a number of different lengths of DNA N ($N = 10$ dotted, $N = 25$ dashed, $N = 50$ solid) and concentrations c ($c = 50$nM black, $c = 500$nM red, $c = 5\mu$M blue). The actual kinetics, upon normalization by N do not change a great deal. Differences only manifest at low $N = 10$, which we attribute to edge effects. These effects lead to quantitative differences, but the same qualitative behavior is observed. The normalization of n_B by chain length N suggests a local process of concentration-enhanced unbinding.

In the analytical results, Equation 10 does not contain any reference to the length of the chain. The numerical results likewise do not include the chain length to be a factor in the matrix k_{ij}, suggesting that the dynamics of these systems is independent of chain length. This is further backed up by the highly local $g(j)$ functions in Figure 2c. Therefore, we expect that the decay in the fraction of binders that remain bound to the chain during an exchange experiment ($\langle n_B\rangle/N$) should decay with the same time evolution $\langle n_B\rangle/N = f(t) \neq f(t,N)$ in a way that does not depend on N. At large N this holds true, though edge effects appear at smaller values of N. This is illustrated in Figure S1, which plots the fraction $\langle n_B\rangle/N$ as a function of time t for $N = 50,20,10$.

$N = 50$ and $N = 20$ are essentially identical (within simulation error). This significantly suggests that, at least at the large N limit, this scaling holds true ($\langle n_B\rangle \sim N$). The time evolution is qualitatively similar, but at $N = 10$ edge effects apparently start to influence results. We anticipate that these edge effects are due to the oscillatory correlation functions shown in Figure 2, which extend a distance of ca. 3 sites away along the chain. This suggests that beads less than 3

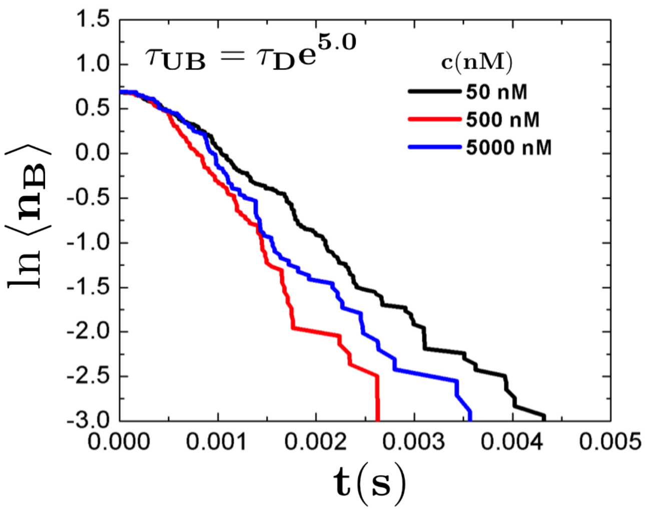

Figure S2. Exchange kinetics ($\ln\langle n_B\rangle$ versus t) for $\Delta\tilde{E}_0 = -5.0$ and $N = 2$, with a number of different concentrations c. c-dependent unbinding is observed, on the same order of magnitude as in Figure S1 and Figure 3 however quantitative differences arise due to chain end effects which dominate at $N = 2$. This limiting case is often used in experimental investigations,(9) and we demonstrate here that c-dependence must be taken into account even in small DNA oligomer investigations.

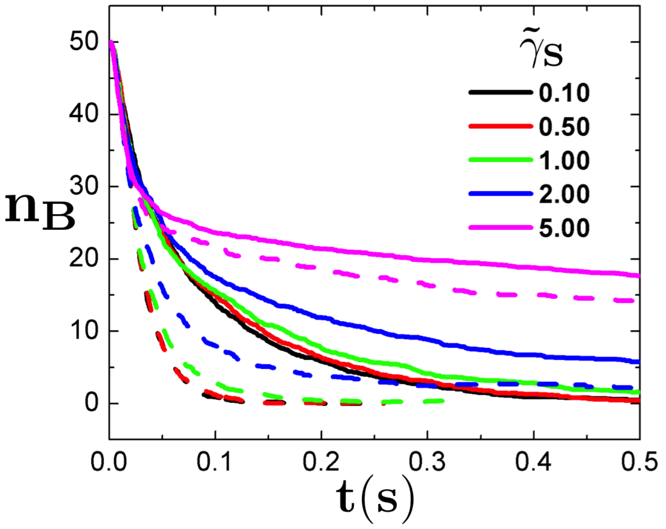

Figure S3. Random and Gaussian-distributed variations in binding energy $\Delta\tilde{E}_0$ are shown for both $c = 5\mu$M (dashed lines) and $c = 50$nM (dotted lines). The random energies have a mean value of $\langle\Delta\tilde{E}_0\rangle = -7.0$ and have a variance of $\tilde{\gamma}_S$. For both concentrations c, the large values of $\tilde{\gamma}_S$ decrease the extent of the exchange with only a small increase in the initial rate of exchange due to the faster-than-usual binders.

indices away from a chain end have different equilibrium and therefore dynamic behavior than long chains. This would alter the quantitative (but not qualitative) picture in a complicated fashion.

The kinetics of DNA-protein binding interactions are often observed using DNA oligomers with sequences that specifically attach to the binding proteins of interest.(9) This results in a situation that in the context of our work is $N = 2$. The derivation of our analytical and numerical theory had long DNA chains in mind, however in principle there is nothing about the shortness of the chain that prevents the same *qualitative* mechanism from applying in this special limiting case. Indeed, the states considered in Figures 3 and 5 only involve 2 binding sites. To illustrate that this concentration-dependent unbinding effect is indeed still relevant, albeit quantitatively altered, we ran a few simulations at $\Delta\tilde{E}_0 = -5.0$ for a number of concentrations c, which are plotted in Figure S2. Clearly, concentration plays a prominent role that must be accounted for in these experiments.

RANDOM BINDING EFFECTS

In experiment, it has been observed that large swaths of binders remain bound for a large amount of time, and do not leave even at long times after the apparent relaxation time scale. These are not homogeneously dispersed; it appears that large numbers of binders are clustered at positions along the chain, an effect which may be due to sequence heterogeneity. In a DNA chain, there are a limited number of permutations of sequence spanning the two dimers in a chain. Nevertheless, it is not clear what the connection is between binding sequence and binding strength is in these systems. Therefore, we investigate the behaviors resulting from a non-constant binding strength and consider a system that represents the non-constant binding extreme situation: a fully randomized binding strength. Such random binding energies are known to have significant effects on the one-dimensional diffusion of protein-binding DNA,(10) and we can explore the possibility of strong effects in this dynamic process as well. We introduce this into the system by considering a binding energy $\Delta\tilde{E}_{UB} = \Delta\tilde{E}_{UB,0} + \xi_{B,s}\tilde{\gamma}_S$ that represents a Gaussian distribution of random corrections to the mean binding strength $\Delta\tilde{E}_{UB,0}$. This is characterized by a magnitude of the random energetic contribution $\tilde{\gamma}_S$ and a set of random numbers $\xi_{B,s}$ that each correspond to a DNA position s and are distributed around $\langle\xi_{B,s}\rangle = 0$ with Gaussian distribution such that $\langle\xi_{B,s}\xi_{B,s'}\rangle = \delta_{s,s'}$.

Figure S3 demonstrates the effect of including a random binding energy at $\Delta\tilde{E}_0 = -7.0$ and two extreme values of c (50nM and 5000nM). Dynamics at both concentrations are essentially unchanged upon small random deviations being introduced ($\tilde{\gamma}_S < 1.0$), however the time scale and long-time limit of the decay both increase significantly when there is a large amount of variance in the binding energies ($\tilde{\gamma}_S = 1.0, 2.0$). This suggests that sequence heterogeneity may be a primary reason for the experimental observations that the decays are not to $n_B = 0$ in Figure 6, which is the main difference between our predictions and the experimental data in Graham, et al.(11)

TRANSFER-MATRIX THEORY FOR DIMER BINDING EQUILIBRIUM

In order to understand the equilibrium properties of these DNA-protein binding simulations (and subsequently the behavior of the experimental systems) we use a transfer matrix calculation of the partition function. This method is well-known in statistical mechanics, and we demonstrate how an

abundance of dimer binding statistics can be obtained upon using these tools. As a simplified representation of such a calculation, we can determine the partition function (and number of bound binders n_B) for a system of monomeric binders. This will yield Equation 2, which can be determined in a number of alternative routes.

To calculate the grand partition function, we must calculate:

$$\Xi = \sum_{\vec{n}_B} e^{\beta \vec{n}_B \cdot \left[\Delta \vec{E}_0 + \vec{\mu}\right]} \tag{4}$$

where we use a vector notation and $\vec{n}_B$ represents the occupation (each component i is 1 or 0 for bound or unbound at index i respectively) state of the system, and $\Delta\vec{E}_0$ and $\vec{\mu}$ are the vectors where the ith components are the binding energy upon binding at i and chemical potential at i respectively. This partition function can be calculated, rather than a direct sum, by a multiplication of matrices $\mathbf{M}_{i,j}$ that represent the conditional probabilities of the state at the index i based on the possibilities of what the state was at index $i-1$. For this system, such a matrix is:

$$\mathbf{M}_{i,j} = \begin{pmatrix} 1 & 1 \\ P & P \end{pmatrix} \tag{5}$$

where $P = e^{-\Delta\tilde{E}+\tilde{\mu}}$ is the contribution to the partition function of having a bound binder at an arbitrary index (we proceed with the homogenous case that $\Delta\vec{E}_0 = \mathbf{1}\Delta E_0$ where all indices have a binding energy ΔE_0) and 1 is the contribution to the partition function of having an unbound position. The matrix provides all the contributions to the partition function at position i given all the contributions at position j. The partition function for a chain of length N is then:

$$\Xi = \vec{\phi}_N \mathbf{M}_{i,j}^{N-2} \vec{\phi}_0 \tag{6}$$

where $\vec{\phi}_N = \vec{\phi}_0 = (1P)$ are the contributions from the first and last positions. This iterative equation is tedious, but as long as N is large the result can be well-approximated by the largest eigenvalue of $\mathbf{M}_{ij}$, λ_0, to the N-th power:

$$\Xi \approx \lambda_0^N \tag{7}$$

The eigenvalue of the above matrix $\mathbf{M}_{i,j}$ for the monomeric binding (Equation S5) is $\lambda_0 = (1+P)$, so for monomer binding we have:

$$\Xi = (1+e^{-\Delta\tilde{E}+\tilde{\mu}})^N \tag{8}$$

The thermodynamic relationship $-k_BT\ln\Xi = G - \mu n_P$ (the value of n_P counts the number of singly plus doubly bound dimers as opposed to the occupied binding sites n_B; the two values are equivalent for monomers but not for dimers) allows the determination of n_B from Ξ:

$$\langle n_P \rangle = \frac{k_BTN}{\lambda_0}\frac{\partial\lambda_0}{\partial\mu} = N(1+e^{-\Delta\tilde{E}_0+\tilde{\mu}})^{-1} \tag{9}$$

which is the result presented in the manuscript.

The same method can be used to determine the much more complicated scenario of dimeric binding. In this situation, the matrix $\mathbf{M}_{i,j}$ is 3×3 and the states are unbound, bound by the first monomer in the dimer, and bound by the second monomer in the dimer. We write this matrix as:

$$\mathbf{M}_{i,j} = \begin{pmatrix} 1 & 1 & 1 \\ P & P & P \\ 0 & P' & 0 \end{pmatrix} \tag{10}$$

where the first row corresponds to the possibility of moving from any state (unbound, first monomer, second monomer) to the unbound state, the second row corresponds to the possibility of moving from any state to the first monomer in a dimer being bound (with the contribution P the same as earlier), and the third row corresponds to the possibility of moving from only the first monomer in the dimer to the second monomer in the dimer (with the contribution $P' = e^{-\Delta\tilde{E}_0}$). P' does not include the chemical potential $\tilde{\mu}$ since the binder has already bound once upon the chain and has already moved from outside the system. An additional contribution of 1 to account for the loss of rotational degrees of freedom upon binding is not included, since the simulation constraints still permit significant rotational freedom even in the bound state. We attribute the slightly non-constant nature of $\tilde{\mu}_0$, which varies by about $k_BT/2$ in our fits ($-\tilde{\mu}_0 = 14.1, 13.4, 13.2, 13.1$ for $c = 0.05, 0.5, 2.5$, and $5.0\mu M$ respectively), to such degree of freedom ambiguities. The largest eigenvalue for this matrix is given by:

$$\lambda_0 = \frac{1}{2}\left[1+P+\sqrt{(1+P)^2+4PP'}\right] \tag{11}$$

which yields the result:

$$\langle n_P \rangle = \frac{k_BTN}{\lambda_0}\frac{\partial\lambda_0}{\partial\mu} = NP\left[\frac{1+\frac{2P'+1+P}{\sqrt{(1+P)^2+4PP'}}}{1+P+\sqrt{(1+P)^2+4PP'}}\right] \tag{12}$$

which is the result in Equation 2.

We note that any number of other similar calculations are possible using this method. We can introduce an artificial field μ_2, for example, that acts only on states of interest. For example, we can calculate the prevalence of singly-bound states using the matrix:

$$\mathbf{M}_{i,j} = \begin{pmatrix} 1 & P'' & 1 \\ P & PP'' & P \\ 0 & P' & 0 \end{pmatrix} \tag{13}$$

where we have included the factor $P''=e^{\tilde{\mu}_2}$ on the components that are characteristic of singly-bound states (i.e. those transfer movements that involve the first monomer on a dimer going to anything other than the second monomer on the same dimer). The largest eigenvalue can be found to be:

$$\lambda_0=\frac{1}{2}\left[1+PP''+\sqrt{(1+PP'')^2+4PP'}\right] \quad \mathbf{(14)}$$

and the number of singly bound states $\langle n_B^*\rangle$ is:

$$\langle n_B^*\rangle=\lim_{P''\to 1}\frac{k_BTN}{\lambda_0}\frac{\partial\lambda_0}{\partial\mu_2}=NP\left[\frac{1+\frac{(1+P)}{\sqrt{(1+P)^2+4PP'}}}{1+P+\sqrt{(1+P)^2+4PP'}}\right] \quad \mathbf{(15)}$$

MASTER EQUATION NUMERICAL THEORY

We presented an abbreviation of the numerical approach to calculating exchange kinetics using the Master Equation representation of the simplifies states of the system. To provide a more thorough picture of how we carried out this calculation, we discuss the development of this theory in more detail.

The starting point for a Master Equation approach is the Master Equation itself:

$$\frac{\partial\phi_i}{\partial t}=\sum_j k_{ij}\phi_j \quad \mathbf{(16)}$$

where the matrix of rate constants k_{ij} has units of $1/time$. We defined the full matrix conceptually in Figure 3 and mathematically in Equation 5. This allowed the writing of an evolution equation upon expanding time evolution using small time increments Δt:

$$\phi_i(t+\Delta t)\approx\phi_i+\Delta t\frac{\partial\phi_i}{\partial t}=\left[\delta_{ij}+\Delta t k_{ij}\right]\phi_j(t) \quad \mathbf{(17)}$$

where the matrix $\left[\delta_{ij}+\Delta t k_{ij}\right]$ can be thought of as an operator that evolves the state at time t, $\phi_j(t)$, to a later time $\phi_j(t+\Delta t)$. We can therefore numerically solve for the exchange process at time $t=\Delta t\times n$:

$$\phi_i(t=n\times\Delta t)=\left[\delta_{ij}+\Delta t k_{ij}\right]^n\phi_j(t=0) \quad \mathbf{(18)}$$

where the initial state is that all the binders are fully bound $\phi_j(t=0)=\delta_{j0}$. In principle, this works for any system so long as k_{ij} is appropriately defined, and the states ϕ_i can be articulated in an unambiguous fashion. This is often practically difficult due to the abundance of independent and interrelated states that can be defined. In our system, we provided a simplified picture that focuses on a minimal representation of ϕ_i such that we can define a relatively straightforward representation of the rate constants k_{ij}, an approach that has found success in similar systems.(6)

To determine the dimers that have separated from the DNA chain and moved outside of the simulation box (and hence become untagged), we must convolute the unbound states with the Green's function $G(0,E;\tau)$ to diffuse to the edge E of the box:

$$\langle n_B\rangle=n_{B,0}-\int_0^t\frac{\partial}{\partial\tau}\left[\delta_{i,5}\left[\delta_{ij}+\Delta t k_{ij}\right]^{\tau/\Delta t}\phi_j(0)\right]G(0,E;t-\tau)d\tau \quad \mathbf{(19)}$$

This equation describes the incremental increase in occupation of state $i=5$ due to the binder processes indicated by Equation S18, and subsequently the binder propagates diffusively (via the Green's function G) to the boundaries of the simulation box. It is this process which dictates the decay of the original bound population of binders $n_{B,0}$. While in a perfectly cylindrical region around the DNA molecule we could, in principle, calculate the exact Green's function since 2-dimensional diffusion is a well-known process.(12) We use an approximate form, since for this aspect of the calculation it is not imperative that we have anything too complicated. For this system, we use the approximation:

$$G(0,E;t)\approx e^{-t/\tau_{DE}} \quad \mathbf{(20)}$$

where $\tau_{DE}=N^2a^2/D$ is the diffusive time scale for a dimer with diffusion constant D to reach a distance of $2Na$ (the distance from the center to one of the faces).

This method, upon quantitative comparison with the simulation results, permits for a much more rapid calculation of the exchange kinetics; instead of iterating through the non-deterministic (i.e. multiple runs are needed to evaluate averages) Langevin equation for ca. $100-900$ species, we are able to iterate through what is essentially 5×5 matrix multiplication. Since the time scales of the experimental results are on the order of 1000s, this results in $1\times 10^{10}-10^{11}$ iterations and is prohibitively long for a Langevin simulation.

REFERENCES

1. Sugimura, S., and Crothers, D.M. (2006) Stepwise binding and bending of DNA by *Escherichia coli* integration host factor. *Proc. Natl. Acad. Sci. USA* **103** 18510-18514.
2. Stella, S., Cascio, D. and Johnson, R.C. (2010) The shape of the DNA minor groove directs binding by the DNA-bending protein Fis. *Genes & Development.* **24** 814-826.
3. Skoko, D., Yoo, D., Bai, H., Schnurr, B., Yan, J., McLeod, S.M., Marko, J.F. and Johnson, R.C. (2006) Mechanism of chromosome compaction and looping by the *E. Coli* nucleoid protein Fis. *J. Mol. Biol.* **364** 777-798.
4. Bell, G.I. (1978) Models for specific adhesion of cells to cells. *Science.* **200** 618-627.
5. Sing, C.E. and Alexander-Katz, A. (2011) Equilibrium structure and dynamics of self-associating single polymers. *Macromolecules* **44** 6962-6971.
6. Sing, C.E., and Alexander-Katz, A. (2012) Force spectroscopy of self-associating homopolymers. *Macromolecules.* **45** 6704-6718.
7. Parsaeian, A., Marko, J.F. and Olvera de la Cruz, M. (2013) Binding-rebinding dynamics of proteins interacting non-specifically with a long DNA molecule. *Phys. Rev. E* **88** 040703(R).
8. Sidorova, N.Y., Scott, T., and Rau, D.C. (2013) DNA concentration-dependent dissociation of *Eco*RI: Direct transfer or reaction during hopping. *Biophys. J.* **104** 1296-1303.
9. Joshi, C.P., Panda, D., Martell, D.J., Andoy, N.M., Chen, T.-Y., Gaballa, A., Helmann, J.D. and Chen, P. (2012) Direct substitution and assisted dissociation pathways for turning off transcription by a MerR-family metalloregulator. *Proc. Natl. Acad. Sci. USA* **109** 15121-15126.
10. Hu, T., and Shklovskii, B.I. (2006) How does a protein search for the specific site on DNA: the role of disorder. *Phys. Rev. E* **74** 021903.

11. Graham, J.S., Johnson, R.C., and Marko, J.F. (2011) Concentration-dependent exchange accelerates turnover of proteins bound to double-stranded DNA. *Nuc. Acids Res.* **39** 2249-2259.
12. McQuarrie, D.A. (2000) *Statistical Mechanics.* University Science, Sausalito.
